# Complex mosaic structural variations in human fetal brains

**DOI:** 10.1101/gr.262667.120

**Published:** 2020-12

**Authors:** Shobana Sekar, Livia Tomasini, Christos Proukakis, Taejeong Bae, Logan Manlove, Yeongjun Jang, Soraya Scuderi, Bo Zhou, Maria Kalyva, Anahita Amiri, Jessica Mariani, Fritz J. Sedlazeck, Alexander E. Urban, Flora M. Vaccarino, Alexej Abyzov

**Affiliations:** 1 Department of Health Sciences Research, Center for Individualized Medicine, Mayo Clinic, Rochester, Minnesota 55905, USA;; 2 Child Study Center and Department of Neuroscience, Yale University, New Haven, Connecticut 06520, USA;; 3 Department of Clinical and Movement Neurosciences, Queen Square Institute of Neurology, University College London, London NW3 2PF, United Kingdom;; 4 Departments of Psychiatry and Genetics, Stanford University, Palo Alto, California 94305, USA;; 5 Human Genome Sequencing Center, Baylor College of Medicine, Houston, Texas 77030, USA

## Abstract

Somatic mosaicism, manifesting as single nucleotide variants (SNVs), mobile element insertions, and structural changes in the DNA, is a common phenomenon in human brain cells, with potential functional consequences. Using a clonal approach, we previously detected 200–400 mosaic SNVs per cell in three human fetal brains (15–21 wk postconception). However, structural variation in the human fetal brain has not yet been investigated. Here, we discover and validate four mosaic structural variants (SVs) in the same brains and resolve their precise breakpoints. The SVs were of kilobase scale and complex, consisting of deletion(s) and rearranged genomic fragments, which sometimes originated from different chromosomes. Sequences at the breakpoints of these rearrangements had microhomologies, suggesting their origin from replication errors. One SV was found in two clones, and we timed its origin to ∼14 wk postconception. No large scale mosaic copy number variants (CNVs) were detectable in normal fetal human brains, suggesting that previously reported megabase-scale CNVs in neurons arise at later stages of development. By reanalysis of public single nuclei data from adult brain neurons, we detected an extrachromosomal circular DNA event. Our study reveals the existence of mosaic SVs in the developing human brain, likely arising from cell proliferation during mid-neurogenesis. Although relatively rare compared to SNVs and present in ∼10% of neurons, SVs in developing human brain affect a comparable number of bases in the genome (∼6200 vs. ∼4000 bp), implying that they may have similar functional consequences.

Somatic mosaicism, the presence of more than one genotype in the somatic cells of an individual, is a prominent phenomenon in the human central nervous system. Forms of mosaicism include aneuploidies and smaller copy number variants (CNVs), structural variants (SVs), mobile element insertions, indels, and single nucleotide variants (SNVs). The developing human brain exhibits high levels of aneuploidy compared to other tissues, generating genetic diversity in neurons (Pack et al. 2005; Yurov et al. 2007; Bushman and Chun 2013). Such aneuploidy was suggested to be a natural feature of neurons, rather than a distinctive feature of neurodegeneration. However, the frequency of aneuploidy in neurons has been debated, with a separate study suggesting that aneuploidies occur in only about 2.2% of mature adult neurons (Knouse et al. 2014). They hence infer that such aneuploidy could have adverse effects at the cellular and organismal levels. Additionally, analysis of single cells from normal and pathological human brains identified large, private, and likely clonal somatic CNVs in both normal and diseased brains (Gole et al. 2013; McConnell et al. 2013; Cai et al. 2014; Knouse et al. 2016; Chronister et al. 2019; Perez-Rodriguez et al. 2019), with 3%–25% of human cerebral cortical nuclei carrying megabase-scale CNVs (Chronister et al. 2019) and deletions being twice as common as duplications (McConnell et al. 2013). Given that CNVs often arise from nonhomologous recombination and replication errors, their likely time of origin is during brain development. However, when CNVs first arise in human brain development has not yet been investigated. The present work is the first to examine this question using clonal populations of neuronal progenitor cells (NPCs) obtained from fetal human brains.

Detection of CNVs in single neurons is challenging, given the need to amplify DNA. Such amplification may introduce artifacts that could, in turn, be misinterpreted as CNVs. In order to address this technical limitation, Hazen et al. reprogrammed adult postmitotic neurons using somatic cell nuclear transfer (SCNT) of neuronal nuclei into enucleated oocytes (Hazen et al. 2016). These oocytes then made sufficient copies of the neuronal genome allowing for whole-genome sequencing (WGS), thus eliminating the need for amplification in vitro. Using this method, they identified a total of nine structural variants in six neurons from mice, three of which were complex rearrangements. However, it is not possible to extend such studies to humans, given the ethical issues involved, besides the technical challenges in obtaining and cloning adult neurons. To circumvent the need of single-cell DNA amplification or nuclear cloning, we examined clonal cell populations obtained from neural progenitor cells from the frontal region of the cerebral cortex (FR), parietal cortex (PA) and basal ganglia (BG) and describe here the discovery and analysis of mosaic SVs in these NPCs (Bae et al. 2018). These clones were sequenced at 30× coverage (much higher than most previous single-cell studies), allowing identification of SVs other than large deletions and duplications as well as precise breakpoint resolution.

## Results

### Complex SVs in fetal brain

**Figure 1.**
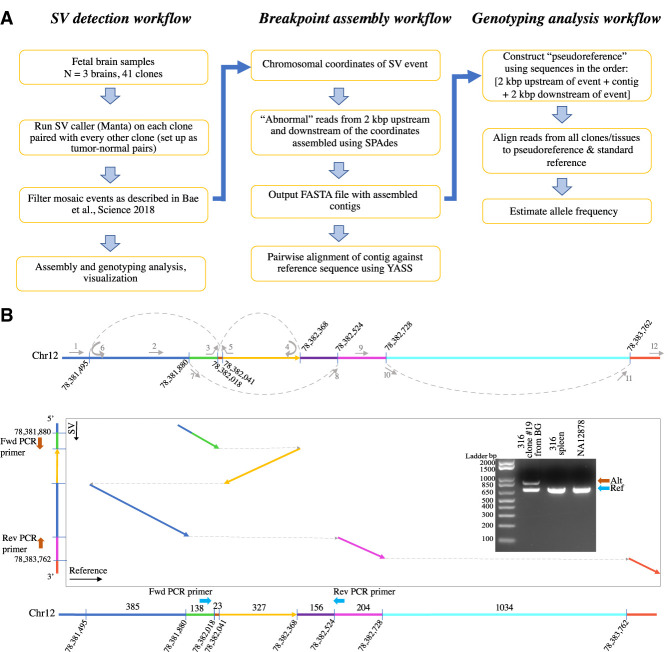
SV detection and analysis workflow with an example of complex rearrangement. (*A*) We used Manta to call SVs in our fetal brain clones. Each clone was compared to all other clones, and germline events were filtered out based on multiple recurrence in the clone-to-clone comparison (Methods). For the resulting high-confidence SVs, we implemented assembly and genotyping workflows as a further in silico validation. (*B*) Example of mosaic complex SV detected. This intra-chromosomal rearrangement on Chromosome 12 (intronic in *NAV3* gene) was detected in subject 316 clone #19 from basal ganglia (BG) and involves two deletions, one inversion, and one duplication. Based on the contig sequence generated from our assembly analysis, we hypothesize a replication model as depicted by arrows in the *top* panel (dashed lines and arcs represent hypothesized replication fork switches). Pairwise alignment of the contig against the reference sequence confirmed the SV and defined the breakpoints of rearrangements (colored arrows in the box represent aligned segments). Using two sets of forward and reverse primers indicated by block blue (Ref) and brown arrows (Alt), polymerase chain reaction (PCR) validated the SV in this clone (results for the second set of primers are represented in Supplemental Fig. S3). Blue arrow: duplication; yellow arrow: inversion; cyan and purple segments: deletions.

We analyzed SVs with a custom workflow that includes SV discovery, local assembly of the allele with an SV, and SV genotyping across multiple samples (Fig. 1A). SVs were called in fetal brain clones (*N* = 3 brains and 41 clones) using Manta (Chen et al. 2016), and somatic events were selected based on clone-to-clone comparisons (Methods). Briefly, each clone was compared against every other clone to call SVs, and calls that were consistently made for the same clone but from different comparisons were retained as true mosaic SVs. For our assembly and genotyping analyses, abnormal reads around each candidate somatic SV were assembled to generate contigs confirming the breakpoints (Methods).

Overall, we identified 32 candidate mosaic SVs across the 41 fetal brain clones. Based on Integrative Genomics Viewer (IGV) (Robinson et al. 2011) visualization for presence in corresponding bulk/tissue or other clones, and overlap with the SV catalog in the Database of Genomic Variants (DGV), we removed 18 likely false positive or germline events (Supplemental Fig. S1) to retain 14 high-confidence mosaic SVs. Among these, five SVs were simple deletions. Of the remaining nine events, some were adjacent and, based on local assembly, we merged them into five complex SVs involving multiple rearrangements. One of these five complex SVs was also detected by CNVnator. Overall, we identified a total of five complex SVs and five simple deletions. We then distinguished four clonal SVs (true mosaic variant) from six subclonal SVs (arising during culture) based on the number of supporting reads and the presence of heterozygous SNPs in the deleted region (heterozygous SNPs are indicative of two haplotypes, while only one is expected for clonal deletions). Breakpoints for each rearrangement were determined by aligning the assembled contigs against the reference genome using YASS (Noé and Kucherov 2005). All four clonal SVs were complex and had multiple rearrangements, while all but one subclonal SV were simple and involved only one rearrangement (Table 1; Supplemental Table S1). Based on germline SV calling, our approach achieved an average of 88% sensitivity across all clones (Supplemental Fig. S2; Methods).

**Table 1.** Summary of detected clonal SVs

SV coordinates	Clone	SV Type	Genomic region
Chr 2: 2,685,870−2,685,883	Brain 320 clone #4 and clone #8 from FR	Deletion + insertion	Intergenic
Chr 15: 98,097,662−98,098,720	Brain 316 clone #3 from FR	Deletion + duplication	LOC101927310 (long noncoding RNA region)
Chr 2: 32,360,001−32,361,600	Brain 275 clone #3 from PA	Deletion + inversion	Intronic in *SPAST* gene (hereditary spastic paraplegia gene)
Chr 12: 78,381,480−78,383,762	Brain 316 clone #19 from BG	Deletion + duplication + inversion	Intronic in *NAV3* gene (neuron navigator)

The most complex SV was an intra-chromosomal rearrangement on Chromosome 12 in subject 316 clone #19 from BG. This SV encompassed two deletions, one inversion and one duplication, with the final rearrangement spanning a length of 1439 bp (Fig. 1B). To explain the identified rearrangements, we hypothesize a replication model of this SV involving four local replication fork switches. Using two sets of forward and reverse primers, we PCR-validated the SV in this clone (Fig. 1B; Supplemental Fig. S3). Sanger sequencing of the diagnostic bands (Supplemental Table S2) confirmed assembled contigs with 100% sequence match. This SV falls in the intronic region of the *NAV3* gene (neuron navigator 3), which is widely expressed in the nervous system, including early development (Fagerberg et al. 2014; Amiri et al. 2018).

**Figure 2.**
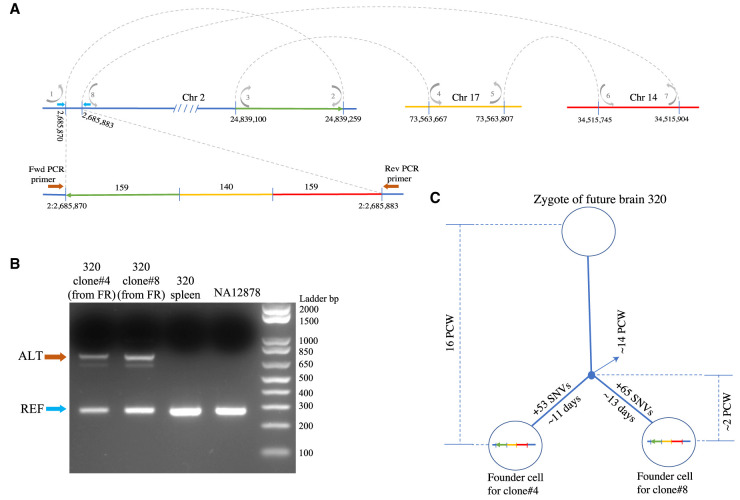
Inter-chromosomal complex SV. We detected an inter-chromosomal complex SV involving Chromosomes 2, 14, and 17 in subject 320 clones #4 and #8 from the frontal cortex. This SV entails a 13-bp deletion on Chromosome 2 into which segments from a farther region on Chromosome 2 (159 bp in length and inverted, green segment), Chromosome 17 (140 bp in length, yellow segment), and Chromosome 14 (159 bp in length, red segment) are inserted. (*A*) Replication model based on the breakpoints supported by the assembled contig is depicted by gray arrows. Location of PCR primers are indicated by blue (Ref) and brown (Alt) block arrows. (*B*) PCR validation confirmed the presence of the SV in both the clones. Blue arrows indicate the REF bands and brown arrows indicate the ALT bands. Both bands were Sanger sequenced. (*C*) Given the occurrence of this complex SV in two clones, and based on our previous estimation that mutations accumulate at the rate 5.1 SNVs per day per neuronal progenitor during neurogenesis (Bae et al. 2018), we expect this event occurs at approximately 14 wk postconception.

One of the complex SVs was inter-chromosomal, involving Chromosomes 2, 14, and 17 in clone #4 and clone #8 from FR of subject 320 (Fig. 2A; Supplemental Fig. S4). This SV entails a 13-bp deletion on Chromosome 2, into which segments from Chromosome 17 (140 bp) and Chromosome 14 (159 bp) are inserted. With PCR, we were able to amplify the entire SV and validate the assembled contigs with 100% sequence match (Fig. 2B). Further genotyping confirmed the presence of the SV in these two clones only (Supplemental Fig. S5A). Given the presence of this SV in two clones, it is likely that the SV arose in the common ancestor cell of these clones. To more precisely time the event, we compared these two clones to each other and identified unique SNVs in each of them (*N* = 53 SNVs unique to clone #4 and *N* = 65 SNVs unique to clone #8). We reasoned that SNVs unique to each clone would have arisen post-divergence of the parent cell into daughter clones. We estimated their time of divergence as roughly 2 wk prior to cell harvesting based on our previous estimation that mutations accumulate at the rate of 5.1 SNVs per day per progenitor (Bae et al. 2018). Since we know the age of the brain, we timed the origin of this complex SV at approximately 14 wk postconception (Fig. 2C).

The remaining two complex clonal SVs were intra-chromosomal and were identified in subject 275 clone #3 from PA and subject 316 clone #3 from FR (Supplemental Figs. S6, S7). These events involved a deletion with inversion spanning 2138 bp and a deletion with duplication spanning 1092 bp, respectively. The former SV was also the one identified from analysis of CNVnator calls and falls in the intronic region of the *SPAST* (spastin) gene, while the latter overlaps with a long noncoding RNA, LOC101927310. As with *NAV3* discussed above, *SPAST* is expressed in early neural development (Amiri et al. 2018). Mutations in the *SPAST* gene have been linked to hereditary spastic paraplegia (HSP), a condition characterized by degeneration of nerve fibers in the corticospinal tracts (Salinas et al. 2008). Using PCR, we were able to validate both SVs in the respective clones (Supplemental Figs. S6, S7), and Sanger sequencing of the bands matched 100% with the assembled contigs.

### Microhomology and size distribution of SVs

**Figure 3.**
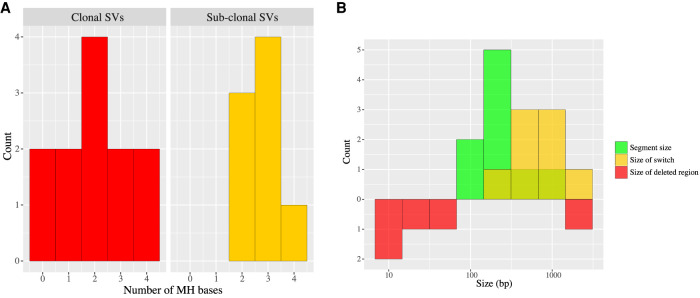
Microhomology and size distribution of SVs. (*A*) Distribution of the number of microhomology (MH) bases detected for mosaic clonal and culture-induced subclonal SVs. (*B*) Distribution of various sizes of the detected clonal SVs. The sizes represented include size of individual segments in the rearranged chromosomal region (green), size of each switch from the replication model (yellow), and the size of deleted regions (red).

Previous studies have reported the existence of short stretches of repeat sequences (∼2–10 bp) known as microhomologies around SV breakpoints (Lee et al. 2007; Zhang et al. 2009; Abyzov et al. 2015). Fork stalling and iterative template switching mechanisms, where the replication fork switches sites of template DNA during DNA replication using sequence microhomology between the switch sites, may lead to complex genomic rearrangements (Carvalho and Lupski 2016), such as the ones we observe here and those previously reported. The existence of such short- and long-distance template switches that occur concurrently suggest a faulty replication process during DNA repair. We thus examined our high-confidence SVs for microhomology around the breakpoints. We observed 2- to 4-bp microhomologies for both the clonal and the subclonal SVs (Fig. 3A). It is hence likely that both clonal and the subclonal events we identified arise as a result of cell proliferation and that the mechanism creating these events is the same. However, clonal events arise in vivo, while subclonal events arise in vitro during clone culturing. The subclonal in vitro events are of interest and deserve further investigation, given the widespread use of stem cell clones and NPCs for human disease modeling.

The absolute size of the SVs we identified (estimated as the length of reference minus the length of alternate alleles) range from 35 bp to 1900 bp for the complex clonal events and from 237 bp to 5609 bp for the subclonal simple deletions (Supplemental Table S1). We observed distinct distributions for the sizes of deletions, replication fork switches (see dashed lines and arcs in Fig. 1B), as well as the replicated (one or more times) fragments (Fig. 3B). Typically, switches were longer (from 253 to 2134 bp) than the replicated fragments (from 243 to 1057 bp) and deleted regions. The latter represent nonreplicated regions, and the largest one we observed was 1891 bp in length, with an adjacent 243-bp inversion (Supplemental Fig. S6). Although the implications of such varied distributions are unclear, it may represent the mechanism(s) involved in replication and how the switches occur during the replication process.

### Genotyping results in tissue/bulk samples

To genotype detected variants, we generated a “pseudoreference” by concatenating the contig with the standard reference sequence around it and aligning reads from all the clones to this pseudoreference (Methods). Owing to the substantial length of the identified SV alleles, reads at multiple positions of the alleles map uniquely. Any such location will genotype the SVs, as opposed to SNVs, for which only reads directly mapping to the position of a variant will genotype it. Therefore, such an SV genotyping approach will be more sensitive compared to SNV genotyping even at standard 30×−40× coverage, as we have in the current study. We applied our genotyping workflow to find evidence of the identified clonal SVs in the tissue/bulk samples of FR, PA, and BG from where the clones were originally derived. As a positive control, we also genotyped the SVs in the respective clones where they were originally identified.

Our genotyping analyses results were consistent with our SV identification results, confirming the presence of the SV in the respective clones where they were identified. However, we found no evidence for these SVs in any of the tissue or bulk samples (Supplemental Fig. S5) and therefore estimated their allele frequencies to be <0.3%. We estimate this from the notion that the number of supporting reads in the clone correspond to ∼50% allele frequency. From this, we extrapolate to the allele frequency if the event is supported by one read. Furthermore, none of the SVs were detected in tissue using droplet digital PCR (ddPCR). Given the amount of input sample used for ddPCR (40 ng of tissue), we achieve a maximum sensitivity of 0.025%, implying that all our variants of interest were below this allele frequency and are hence present in less than one in 4000 cells in the tissue. These results indicate that such SVs most likely occur relatively late in embryonic development, perhaps post-neural tube formation, consistent with our estimate based on the inter-chromosomal SV occurring in two clones.

### SVs in the adult human brain

Given the occurrence of such complex SVs in fetal brains, we hypothesized that similar events might exist in adult brains as well. To this end, we applied our SV calling and clone-to-clone filtering approach to the single nuclei data from adult neurons (Lodato et al. 2015; Sanchez-Luque et al. 2019). In the Lodato et al. study, single cortical neuronal nuclei were isolated from the prefrontal cortex (PFC) of postmortem human brain tissue (*N* = 3 brains, 36 single nuclei) and amplified by multiple displacement amplification (MDA), followed by WGS at a coverage similar to that of our fetal brain clones. In the study by Sanchez-Luque et al., single neuronal nuclei from the hippocampus (HIPP) of a postmortem donor were amplified by MDA and whole-genome-sequenced at ∼47× coverage. We worked with these data sets because of long amplified fragments by MDA of ∼50 kb and high sequencing coverage. Data from similar studies (McConnell et al. 2013; Cai et al. 2014; Knouse et al. 2016) were not suitable for our analysis, given their shallow sequencing coverage and shorter amplicon sizes (∼1 kb).

Overall, in the 36 single nuclei from PFC, we called 17–28 deletions and 71–207 duplications using our cell-to-cell filtering approach. Additionally, Manta detected ∼23,000–31,000 break-ends (BNDs, which are one-sided inversions) per brain across all cell-to-cell comparisons (Supplemental Fig. S8). Given the frequent incidence of chimera artifacts that occur in MDA (Lasken and Stockwell 2007), most of these calls (deletions, duplications, and BNDs) in the PFC data set likely represent false positives. In line with this, we observe that fetal brain clones amplified by MDA (*N* = 3 clones from brain 316) had a large number of BNDs (8063 BNDs in MDA-amplified clones vs. 196 BNDs in others). No deletion or duplication calls passed our filtering criteria in the HIPP data. This was perhaps in part because the amplified cells in this data set were not subjected to the same pre-WGS selection as the PFC data and, as a result, had higher allelic dropout rate and overall less balanced amplification of two haplotypes (Supplemental Fig. S9).

Finding no confident CNVs in the adult brain samples is consistent with previous reports that CNVs can be detected in human brain neurons using GenomePlex, PicoPlex, and Strand-Seq amplification methods that have lower amplification noise compared to MDA, but can only detect megabase-scale events (McConnell et al. 2013; Cai et al. 2014; Chronister et al. 2019). We therefore focused on the strategy of finding complex mosaic SVs by overlapping all SV calls from each cell within 1000 bp of each other. In both PFC and HIPP data sets, we frequently observed distinct combinations of adjacent one-sided inversion with duplication in multiple neurons (Supplemental Fig. S10). We interpreted such SVs as likely false positives, since they resemble the most frequent chimeric artifact of MDA. Further, replication models for such events would imply reversal of the direction of replication fork propagation, indicating incomplete chromosome copying, which is not seen in the cells.

**Figure 4.**
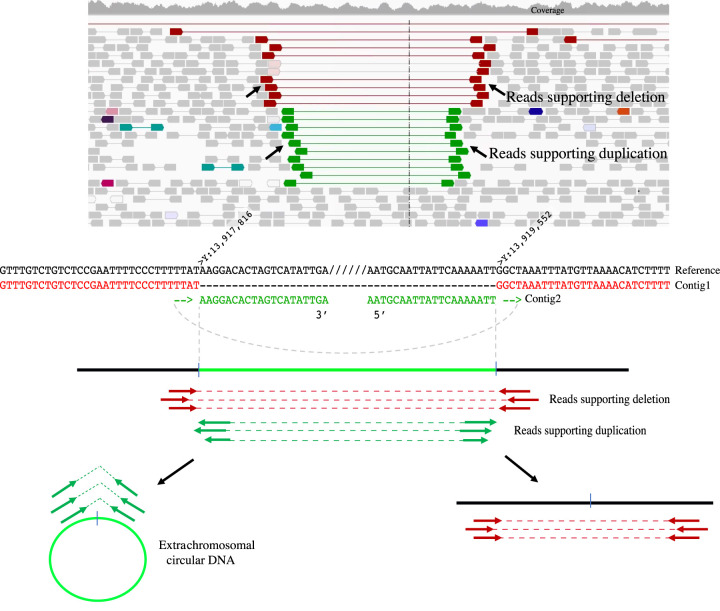
Extrachromosomal circular DNA read support and illustration. We detected a deletion and duplication event in brain B, neuron #12 in the single nuclei data from adult neurons (Lodato et al. 2015). *Top* panel shows a screen-capture of the region in Integrative Genomics Viewer along with the deletion (red) and duplication (green) supporting reads. Reference sequence around the breakpoints and contigs generated from assembly analysis supporting both deletion (red letters) and duplication (green letters) are represented in the *middle* panel. The *bottom* panel illustrates the hypothesis that, given the presence of deletion and duplication supporting reads at the same breakpoints, this event represents an extrachromosomal circular DNA that arises from the deleted region in the reference sequence.

The only two somatic SVs that did not look like obvious artifacts in the single-nuclei data were a deletion and duplication on Chromosome Y in brain B, neuron #12 from the PFC data set (Fig. 4). The two events had reciprocally matching breakpoints, and hence, we hypothesize that they represent a single event where an extrachromosomal circular DNA arises from the deleted region. This event falls in an intergenic region and we did not observe any microhomologies at the breakpoints. We further hypothesize this event to involve two double-stranded breaks repaired by the nonhomologous end joining (NHEJ) mechanism. The repair mechanism could connect them in such a way leading to the formation of a circular DNA arising from the deleted region and a linear sequence. An alternative possibility is that these SVs originate from unequal cross-over between the two haplotypes of a chromosome. However, given that this event occurs on the haploid Chromosome Y (and not in pseudoautosomal regions), we rule out this possibility.

## Discussion

Recent single-cell whole-genome studies (scWGS) have begun to understand and unveil the timing of origin of SNVs in human brains. However, the developmental stage at which SVs first arise in tissues is yet to be investigated. In this study, we identified four somatic SVs in 41 clonal cell populations derived from neuronal progenitor cells, using a paired-end- and split-read-based approach. We found four kilobase-scale complex rearrangements, including one inter-chromosomal SV that occurs in two clones. Based on the presence of unique somatic SNVs in each clone, we timed the origin of this SV at mid-neurogenesis, consistent with a recent report in fetal mouse NPCs showing increased CNV prevalence during mid-neurogenesis (Rohrback et al. 2018).

The approach we used to identify complex SVs in fetal brains is clonal amplification of neuronal precursors, which avoids the artifacts of single-cell DNA amplification. This is a similar strategy to that used in mouse mitral and tufted neuronal subtypes (MT neurons) (Hazen et al. 2016). However, while the rearranged fragments in mice MT neurons ranged between 7 bp and 1.4 Mb, we observed sizes between 35 bp and 1.9 kb for the human fetal brain clonal SVs. This difference in size could be a result of the difference in the developmental stage of the neural cells studied. MT neurons were derived by reprogramming adult postmitotic neurons from mice between ages 3 wk and 6 mo, whereas human fetal brain NPCs were proliferative precursors not yet differentiated into neurons and obtained 15–21 wk postconception. Therefore, the differences in the range of sizes observed between the two studies could be attributed to the differences in the cell type and developmental stage. Further, interspecies and mutability differences may also contribute to the difference in SV sizes.

Several neuronal scWGS studies have reported megabase-scale CNVs, ranging in size between 2 and 159 Mb (Gole et al. 2013; McConnell et al. 2013; Cai et al. 2014; Knouse et al. 2016; Chronister et al. 2019; Perez-Rodriguez et al. 2019). Given their use of different sample preparation and amplification methods as well as informatic analysis workflows, these studies report variable findings, ranging between 0.2 and 3.4 CNVs per cell, affecting between 5% and 30% of the neurons. Further, Chronister et al. reported a negative correlation between individual age and CNV neurons—fewer CNVs per neuron were observed in aged individuals compared to younger individuals. Very occasionally, such CNVs were shared by two neurons, suggesting a developmental origin and clonal sharing; the vast majority, however, were private to a single neuron (Cai et al. 2014; Knouse et al. 2016; Perez-Rodriguez et al. 2019). Combined with our evidence that we did not observe large-scale CNVs in fetal brain, this indicates the possibility that CNVs arise during later stages of neurogenesis, that is, late fetal stages or even after birth, in mature neurons rather than dividing progenitors. This, in turn, suggests that DNA replication-related mechanisms must exist to protect neuronal progenitors from such large scale events. Indirectly, the data implicate different mechanisms by which large CNVs arise in more mature cells. Namely, postmitotically, they are likely to result from faulty DNA repair, as observed in the case described here of circular DNA. Finally, we cannot exclude the possibility that cells that have acquired large CNVs during neurogenesis may not be amenable to clonal expansion, which could also imply negative selection for such events in proliferating progenitors in vivo.

Although we did not find complex SVs in the single-nuclei data from adult neurons, given the relatively small number of clones and single cells analyzed, the results are consistent with those from fetal brain in terms of the rarity of such SVs (four out of 41 samples in fetal brain, 0 out of 36 in adult brain; Fisher's exact test 2-tailed *P*-value: 0.1173). Another possible reason why we were able to identify complex SVs in fetal brain but not in adult brain neurons could be the cellular composition of the collected samples. For example, a block of fetal brain tissues is composed of cells of diverse clonal origin given the intermixing of diverse progenitor cell types. However, in the case of adult neurons originating from a single biopsied site, most of these mature cells arise from a few progenitors that locally expand, and hence they don't have a diverse origin comparable to that of fetal progenitors. Therefore, it is possible that these single cells and derived lineages of cells do not carry any somatic SV as a result of this reduced sampling. In vivo clonality differences between the biopsied samples from adult and fetal brains could thus contribute to the difference in the rate of detected complex SVs. Moreover, previous MDA single-neuron studies have reported allelic and locus dropout rates of 10% or higher (Evrony et al. 2012,^2015^; Lodato et al. 2015; Zhou et al. 2020). Such loss of one or both alleles at a locus during amplification, as well as nonuniformity in amplification, results in reduced sensitivity of single-cell data in identifying structural rearrangements.

In one of the adult neurons, we identified a circular DNA and a reciprocal chromosomal deletion. Previous studies have reported the presence of several circular DNA events in healthy human somatic tissue and blood (Kumar et al. 2017; Møller et al. 2018). Our finding demonstrates that circular DNAs can be detected at the single-cell level. Furthermore, to our knowledge, this is the first report of a circular DNA with reciprocal deletion detected in any tissue.

Recent WGS studies have reported SVs in other tissue types, including skin fibroblasts and adult stem cells from small intestine, liver, and colon. In 10 clonal fibroblast cell lineages, 2–22 somatic CNVs per clone in the size range 1030 bp–88 Mb, and 1–24 somatic structural changes per clone, in the size range 129 bp−66 Mb, were detected (Saini et al. 2016). In clonal organoids derived from adult stem cells (ASCs), deletions ranging in size from 91 kb to 2 Mb were detected in four out of 14 ASCs from the small intestine (Blokzijl et al. 2016). In the same study, deletions ranging in size from 142 kb to 1 Mb, as well as a complex translocation involving three chromosomes and a trisomy of Chromosome 13, were identified in four out of 15 colon ASCs. Further, chromosomal gains ranging in size between 250 kb and 90 Mb and a 500-kb deletion were identified in four out of 10 liver ASCs. SVs detected in our study are significantly less frequent and smaller in size (four out of 41 clones, ∼1−2 kb in size). Again, the tissue of origin and age of the cell could contribute to differences in size and frequency of somatic structural changes, with older adult cells harboring larger SVs/CNVs at higher frequencies. In this regard, we would also like to note that long-read sequencing techniques such as those from Oxford Nanopore Technologies (Jain et al. 2016) and Pacific Biosciences (Eid et al. 2009) are likely better suited for SV identification (Sedlazeck et al. 2018; Mahmoud et al. 2019) and may enable detection of a larger spectrum of SVs present in single cells.

On analyzing the genomic regions of the fetal SVs, we observe that one of the SVs falls in an intergenic region (Chr 2: 2,685,870−2,685,883), one falls in a long noncoding RNA region, LOC101927310 (Chr 15: 98,097,662−98,098,720), and the remaining two SVs fall in the intronic regions of *SPAST* (Chr 2: 32,360,001−32,361,600) and *NAV3* (Chr 12: 78,381,480−78,383,762) genes. *SPAST* encodes a protein called spastin, which is enriched in the distal axons and branches of postmitotic neurons (Errico et al. 2004), and *NAV3* encodes neuron navigator 3 that is predominantly expressed in the nervous system. Mutations in the *SPAST* gene have been linked to HSP, a progressive disabling disorder caused by a length-dependent dying-back spinal cord axonopathy. Hence, two of the four SVs we identified overlap with genes that are expressed in the brain (including fetal brain), while one overlaps with a long noncoding RNA region. In line with these observations, large transcription units have been previously identified as predictors of CNV hotspots and suggested to drive locus instability, given their susceptibility to fork failure (Wilson et al. 2015).

We previously reported a total of 6288 mosaic SNVs in the above analyzed clones. Although SNVs are collectively more numerous, the SVs we detected here affect a similar number of bases in the genome, that is, ∼4000 bp. It is therefore possible that, although rarer than SNVs, the SVs we identified here may have comparable functional consequences.

## Methods

Human fetal brain samples from frontal cortex, basal ganglia, and parietal cortex of subjects 316, 320, and 275 were obtained, processed, and sequenced at 30× coverage as previously described (Bae et al. 2018). For subjects 316 and 320, WGS data of spleen, a nonbrain bulk sample was obtained, while for subject 275, bulk tissue culture was used. We included seven additional clones apart from the ones used in our previous study (total *N* = 41 clones). We had previously excluded these clones because we observed a shift from the 50% allele frequency distribution of variants detected in common between four variant callers: MuTect (Cibulskis et al. 2013), SomaticSniper (Larson et al. 2012), Strelka (Saunders et al. 2012), and VarScan (Koboldt et al. 2012). Since SVs are less frequent compared to SNVs, with the goal of finding more SVs, we included additional samples in the current study for which the major clone in the clonal population had an allele frequency of at least 30%. Binary alignment mapping (BAM) files were generated by aligning raw reads to the reference genome (1000 Genomes Project, human_g1k_v37.fasta) using BWA-MEM v0.7.8 (Li 2013) and post-processed to perform marking of duplicate reads, local indel realignment, and base quality score recalibration using the Genome Analysis Toolkit (GATK v3.4.0) (McKenna et al. 2010). Detailed alignment and post-processing steps are described in Bae et al. (2018). On the resulting BAMs, we ran CNVnator v0.3.3, Manta v1.5.0 (Chen et al. 2016), and DELLY v0.7.8 (Rausch et al. 2012).

For CNVnator, we compared each clone against the respective bulk/tissue sample to identify deletions and duplications. CNVnator utilizes a read-depth approach for CNV discovery and genotyping. CNV calling criteria were defined, and manual inspection of calls was performed as described previously (Abyzov et al. 2012). Briefly, bin size of 200 bp was used for CNV calling. We then removed all calls within 1 Mb of gaps in the reference genome and checked for the read-depth difference between bulk and clones for deletions and duplications using the following criteria: for deletions, the average normalized copy number estimates from read depth of at least 1.6 in tissue and no more than 1.4 in clone, a difference between the two of 0.5, and 60% of other clones having at least 1.6 copy number; for duplications, average normalized copy number estimates from read depth of at least 2.4 in tissue and greater than 2.6 in clone, a difference between the two of 0.5, and 60% of same sample clones having copy number below 2.4. All calls were smaller than 3 kb in size, and such small regions are subject to coverage variability due to randomness and repeat content affecting read mappability. Upon visual inspection of read-depth tracks across all clones and repeat content of each call, only one CNV was deemed as mosaic, which had an inversion adjacent to it (classified as a complex SV).

Next, we ran Manta in the somatic configuration mode with tumor-normal pairs to call SVs. Manta uses paired end and split read evidence to detect SVs. We compared each clone against every other clone and filtered for somatic events from these clone-to-clone comparisons as described previously (Bae et al. 2018). Since breakpoint resolution may not be accurate in SV calling, we merged calls that exhibit 50% reciprocal overlap using BEDTools v2.26.0 (Quinlan and Hall 2010). Manta results were then post-processed to calculate the explanation score for each SV call, and calls with score greater than 0.8 were filtered as somatic. These filtered somatic events were further vetted using our assembly and genotyping analysis workflows as well as visualization using IGV v2.3.92.

Furthermore, we also performed SV calling using DELLY (Rausch et al. 2012) and filtered for somatic events from clone-to-clone comparisons. The four clonal SVs (Table 1) were also called by DELLY. However, DELLY called additional events with distinct size ranges: 500–1000 bp and 2–93 Mb (Supplemental Fig. S11). Upon further inspection of those events in IGV and in other clones, we observed that all of them are likely false-positives, given their presence in bulk/multiple clones, lack of read support based on read-depth plot visualization, or overlap with the SV catalog in DGV. Combined with evidence from previous studies reporting Manta's high sensitivity (Zarate et al. 2018; Chen et al. 2019), we primarily focus on Manta calls, with DELLY providing additional support for our confident call set (Methods).

### Assembly and genotyping analysis workflow

We performed in silico validation of filtered somatic SVs using our assembly and genotyping workflow (Fig. 1A). For each identified SV, our workflow first extracts “abnormal reads” from a 2-kb window on either end of the event from the alignment file (BAM). Abnormal reads include discordant ones (insert size is greater than expected average insert size), soft-clipped reads (>30 bases soft-clipped), and reads with their mates mapping to a different chromosome. The extracted reads are then assembled using SPAdes v3.13.0 (Bankevich et al. 2012) to generate contigs. We then performed pairwise alignment of the contig against the reference sequence using YASS. As a further validation step, we also performed genotyping analysis for each SV in all the clones. To this end, we first construct a “pseudoreference” by concatenating the assembled contig to 2-kb flanking sequences from the reference genome. If homozygous SNPs were present in the pseudoreference sequence, we reverted them back to the original base in the reference sequence. This step ensures that BWA-MEM does not artificially map reads to SV alleles because of the difference in homozygous SNPs with the reference. Reads from all the clones and bulk tissues were then re-aligned to this pseudoreference to calculate allele frequency based on uniquely mapped reads, which will be greater than zero in clones or in the tissue where the event is present.

### PCR and capillary Sanger sequence validations

SVs were analyzed with PCR and subsequent capillary Sanger sequencing. Primers targeting the predicted SV allele were designed using MacVector software (version 17.0.5, MacVector, Inc.). All primer sequences were tested for their uniqueness across the genome using the in silico PCR tool from the UCSC Genome Browser. A human control DNA (NA12878, Coriell Institute) was used as reference sequence. A temperature gradient PCR was performed using the control DNA for each primer pair to optimize the annealing temperature of the reaction. PCR amplifications were performed in a Bio-Rad C1000 Touch thermocycler in 50–100 μL reactions containing 50–100 ng of template DNA. TaKaRa LA (TaKaRa Bio USA, Inc.), a high-fidelity long-range polymerase, was used for predicted PCR amplicons longer than 1 kb. PCR reactions were performed following the manufacturer's recommendations. Briefly, initial denaturation at 94°C for 1 min, followed by 30 cycles of denaturation at 98°C for 10 sec, annealing temperature as determined in gradient assay for 15 sec, extension at 68°C for 2 min, followed by a final extension at 72°C for 10 min. Thermo Fisher Scientific Phusion High-Fidelity DNA Polymerase was used for amplicons under 1 kb. As recommended by the manufacturer, the cycling conditions were: an initial denaturation at 98°C for 30 sec, followed by 30 cycles of denaturation at 98°C for 10 sec, annealing temperature as determined in gradient assay for 30 sec, extension at 72°C for 30 sec, followed by a final extension at 72°C for 10 min. PCR reactions were fractionized in 2% (for amplicons amplified with Phusion High-Fidelity) or 1% (for amplification with TaKaRa LA) agarose gel containing 0.1 µg/mL ethidium bromide for 20–45 min at 100V. Fragments were visualized using UV-fluorescence, reference and alternative bands were excised from the gel, and the DNA was purified using a QIAquick gel extraction kit (Qiagen) following the manufacturer's instructions. Fluorescent sequencing was carried out on an Applied Biosystems 3730 capillary instrument.

### Digital PCR validation in primary tissue and clones

Primers for ddPCR were designed to amplify amplicons between 60 and 200 bp surrounding the SV breakpoint-junction site. Primers’ GC% content was limited to 50%–60%. FAM-conjugated probes were designed to hybridize directly onto the predicted breakpoint-junction sequences. Probes’ GC content was limited to 30%–80%, their size was limited to 25 bp, and a starting G at the 5′ end was avoided. Repeats of four Gs or Cs were avoided in both primers and probes. A primer pair and VIC-conjugated probes targeting the human *RPP30* allele were used as a reference. All ddPCR assays were run on a Bio-Rad QX200 Droplet Digital PCR system. Assays were performed as previously described (Hindson et al. 2013) and following Bio-Rad recommendations. Briefly, 20 ng of genomic DNA was used for each replicate well, primers/probe ratio final concentration was 900 nM/250 nM. No-template control wells were included in all assays to monitor for contamination. NA12878 gDNA was used as a negative control for all assays. The optimal annealing temperature was determined by running a gradient ddPCR assay for each primers/probe reaction. Results were analyzed using the Bio-Rad QuantaSoft software. The allele frequency of SVs and the corresponding 95% confidence interval were calculated according to Dube et al. (2008).

### Estimation of discovery sensitivity

We estimated the sensitivity of our SV discovery approach by comparing WGS data from each clone to that of an unrelated cell line (NA12878) and calculating the fraction of SVs called from such a comparison that are present in our defined “truth set.” To this end, we first compared tissue/bulk samples from each subject to NA12878 to detect SVs. Among the SVs called from this comparison, we defined the “truth set” as those calls that were found independently (but overlap 50% reciprocally) in two different bulk samples of each respective brain. Lastly, we calculate the sensitivity of our SV calling approach by estimating the fraction of SVs from each clone-to-NA12878 comparison present in the truth set.

## Data access

All raw sequencing data generated in this study have been submitted to the NIH National Institute of Mental Health (NIMH) Data Archive (https://data-archive.nimh.nih.gov) under collection ID #2330, study ID #496, and DOI: 10.15154/1410419.

## Competing interest statement

The authors declare no competing interests.

## Supplementary Material

Supplemental Material
