# Supplementary material for: Complex mosaic structural variations in human fetal brains
Source: Genome Res. 2020 Dec;30(12):1695–704. doi: 10.1101/gr.262667.120 (PMC7706730; doi:10.1101/gr.262667.120)
Supplement: Supplemental Material [file supp_30_12_1695__index.html]

Complex mosaic structural variations in human fetal brains — Complex mosaic structural variations in human fetal brains — Supplemental Material 

# Complex mosaic structural variations in human fetal brains

## Supplemental Material

- Supplemental\_Files.docx
- Supplemental\_Table\_S2.xlsx
