# Supplementary material for: Complex mosaic structural variations in human fetal brains
Source: Genome Res. 2020 Dec;30(12):1695–704. doi: 10.1101/gr.262667.120 (PMC7706730; doi:10.1101/gr.262667.120)
Supplement: Supplemental Material [file supp_gr.262667.120_Supplemental_Files.docx]

**Supplemental information**

**Complex mosaic structural variations in human fetal brains**

Shobana Sekar^1^, Livia Tomasini^2^, Christos Proukakis^3^, Taejeong Bae^1^, Logan Manlove^1^, Yeongjun Jang^1^, Soraya Scuderi^2^, Bo Zhou^5^, Maria Kalyva^3^, Anahita Amiri^2^, Jessica Mariani^2^, Fritz J. Sedlazeck^4^, Alexander E. Urban^5^, Flora M. Vaccarino^2^*, Alexej Abyzov^1^*

Supplemental Figure S1: Example of a false positive germline SV excluded after visualization.

Supplemental Figure S2: Sensitivity analysis of our SV calling approach.

Supplemental Figure S3. Second set of PCR primers for complex event on chr12 in brain 316 clone #19 from BG.

Supplemental Figure S4: Complex clonal SV detected in brain 320 clone #4 and clone #8 from FR.

Supplemental Figure S5: Genotyping analysis results for the four clonal SVs.

Supplemental Figure S6: Complex clonal SV on chr2 detected in brain 275 clone #3 from PA.

Supplemental Figure S7: Complex clonal SV on chr15 detected in brain 316 clone #3 from FR.

Supplemental Figure S8: Distribution of various SV types resulting from all-to-all comparisons in fetal and adult brain samples.

Supplemental Figure S9: Variant allele frequency distribution of germline heterozygous SNPs from single nuclei data.

Supplemental Figure S10: Example of a one-sided inversion and duplication event detected systematically in multiple neurons.

Supplemental Figure S11: Size distribution of SV calls from DELLY.

Supplemental Table S1: Summary of clonal and sub-clonal events detected using our custom SV detection workflow summarized in Fig. 1A.

**Supplemental Figure S1:** **Example of a false positive germline SV excluded after visualization** (Chr6:53,273,884-53,274,310). Following clone-to-clone comparison and explanation score calculation, 32 mosaic SVs were visually inspected and checked in multiple clones; 18 SVs were then excluded given their occurrence in multiple clones and/or in the tissue/bulk samples. In the above example, we exclude this SV based on its presence in the corresponding tissue. Grey segments represent mapped reads (MAPQ > 0) and red, green and blue lines in the reads represent soft-clipped fragments.

**Supplemental Figure S2: Sensitivity analysis of our SV calling approach.** Based on our germline SV detection strategy for sensitivity estimation (Methods), we achieved an average sensitivity of 88% across the 3 subjects. Each dot represents a clone.

**Supplemental Figure S3. Second set of PCR primers for complex event on chr12 in brain 316 clone #19 from BG.** Assembled contig and sequence microhomologies were as below:

>Assembled contig (reverse complement)

CTAAAGAGGCATGGCTTGAGATTTTGGAATATGCTGTTTGACTTTAGAAACTGTGAGTATTCTCCTTCCAAGGAGACATCTGTGGATATTCTGTAGCACACAGTATCTCCAATGTTATTATGCATAGGAATCACCTGCGGATTCTGTTTAAATGAAGATTCAAATTCAGTAAGTCTGAGATGGGATCTGAGGTTCTATATTTCTAACAAGCCTACTAAAAGAAGTATGTGATCATCTTTCTAGTAAACTGGAATATCAAGGGGCTGCTTTTTATTTTTCTCAAGCATGGAAGGGAAGAAGAGAAAACTATACACAGTCAACTATTTTTAAAGTATGGAATTCTTCTGGTGATCAAAACACAGCAGGATCCAAACTGCCAGAGCTTCCTATTTCAGCTTCAGTGTAAATCCTCATGTGGAGTTATAAAATCCATTTATTGTAAGGAAAGAAAGCTTTTTAAGGTAAACAGGGCCGAATCTGTGGTTACACATTTTTACTAATCACTACAGCCTCACTACTCAAAATGTGGTCTGTAGACAGATACTTAACCTGATTAATTACTGCACCATCAAGATACAGTATTCAAGACATATCGAGATATTCAGAAAGGGGATTCTGAAGTTTAGGATTGAGATTGGGGTTTGAAATATACATTTGCTATACAAGAAGGAAGGAGTCGATTATATCTGGGATTATAATTTTTAGAATATTTATGGTAACAGCGTAAAATTTTGTGGGTGGTGACAGTGAGTGAACCTGTATTTGGGAAGCAGTAGTAATAGCTGCAGATGTGGCTGTTGTGTCAGGAACGCTAAAATGATGAAGTCTGTAAGAAGGAGGCTGCTGACATCTAAAGAGGCATGGCTTGAGATTTTGGAATATGCTGTTTGACTTTAGAAACTGTGAGTATTCTCCTTCCAAGGAGACTCAGTTTATTTTCCCCCATTGCCTGAGAGCTGTGTCTACATGTCACCTGTGTCTGTTTTACTAGGACTCGGCAAGTTGGCTGTGCTTACTCACCTGGTTTGTTACTAATATGTTATAAACTTGAAACCTGGGAAATGGTATTACAAGCACTGTGGAGGATAAAATGCTTTTCTGCTGTCCAGTGAGGCATAGACAATAATTCCACTATTGATTATTTCAAATCAGCTATTCACCTCAATTTATGTAAAACAAATTAAGTATATATCTGTGAAAGATAGAGTTTATTTTTAATATGCTTCCAATGTTATTCAAACACCAGGGGTTTAGTCTAGGTCCTGCTCTCTGTTGCACAGAAAGCCAATCACTAAAATAACAAGAATTGCCTAGGGAGAAGGCTTTACTCAGTTGCTGCAGCTGACAAGAATGGGAGATCAGTCTCAAATTCACCTCCCCAATTAACTAAAACTGGGGGTTTATATAGTGGGGAAGGCATGTGGCTACATGCAGAAAAGCAGGAATTAGGGAAAGGTAAGGAAGAAAAGTTGGTC

First switch

TGGGATCTGAGGTTCTATATTTCTAACAAG**CC**TACTAAAAGAAGTATGTGATCATCTTTC CONTIG

TGGGATCTGAGGTTCTATATTTCTAACAAG**CC**ccaggtga LEFT 78,382,018

cattgtggat**CC**TACTAAAAGAAGTATGTGATCATCTTTC RIGHT-REV 78,382,368

Second switch

ACTACAGCCTCACTACTCAAAATGTGGTCTGT**AGAC**AGATACTTAACCTGATTAATTA CONTIG

ACTACAGCCTCACTACTCAAAATGTGGTCTGT**AGAC**caac RIGHT-REV 78,382,041

ttagg**AGAC**AGATACTTAACCTGATTAATTA LEFT 78,381,495

Third switch

TTAGAAACTGTGAGTATTCTCCTTCCAAGGA**GAC**TCAGTTTATTTTCCCCCATTGCCTGA CONTIG

TTAGAAACTGTGAGTATTCTCCTTCCAAGGA**GAC**atctgt LEFT 78,381,883

tttct**GAC**TCAGTTTATTTTCCCCCATTGCCTGA RIGHT 78,382,528

Fourth switch

ATGCTTTTCTGCTGTCCAGTGAGGCATAGACAA**TAAT**TCCACTATTGATTATTTCAAAT CONTIG

ATGCTTTTCTGCTGTCCAGTGAGGCATAGACAA**TAAT**gttaaga LEFT 78,382,727

aagctcc**TAAT**TCCACTATTGATTATTTCAAAT RIGHT 78,383,767

| Switch | MH bases | # of MH bases |
| --- | --- | --- |
| Switch1 | CC | 2 |
| Switch2 | AGAC | 4 |
| Switch3 | GAC | 3 |
| Switch4 | TAAT | 4 |

**Supplemental Figure S4: Complex clonal SV detected in brain 320 clone #4 and clone #8 from FR.** Assembled contig and sequence microhomologies were as below:

>Assembled_Contig_for_320_FR4_FR8

TGAGAGGCACTGAAGAGCAGGTAGCAGATACAGACTTGAGTTGTTTATCCACATCCCTTGGAGCATCCTGGAGTTTGACCTTCAGCATGTGTGAGTGGAATAGCCCTGCAACCTCATACTCCACTTGAGAAAGCCTCAGCCTTGGTTACTTATTTCCCAGCATACAAACAAATGCACATGCACGCCCACATATGTGGCATGCACAATGGAAATGTCGGCACGTTCACTTGCTGATGGAAATGACTTAGAGTGGGCACAGTTAATAACTAGGAAGGAAGAGACACGTACTTCAGTGACAGTTTCCCCAGATTGCGAGAGGATGTGTCCAGTGAGAGTGGGAGTCACTGGTTCTGGATGGACCCACGGACGGTTCATCTCTAGTCGAAGTCAGCCGCAGGCAGGAAGGCAGAGGGCAGGGTGCAGAGGCTGGCGGGGGAAGGGCGGTGGAAAGGCCTGGCAGGTTCCAACATTGGAGAAAGATGGAAAGAAATCTCAACTATCAAATCCATACAAGAGCAACAAACGGTATCAAATTAAAACTATGTACTATGGTAACTATGTAACAAATTATTTTTCATCATCTGTGAAAAAGGATGGTAGGAAAAGGGAAATGTGCAAATAGCAGCTGAGATCGCACCACTACACTCCAGCCTGGGTGACAGAGCAAGACTCCATCTCAAAAAAAAAAGAGAAAAAAAAGACAGGGCACAGTGGCTCATGCCTGTAATCCCAGCATTTTGGGAGGCCAAGGTGGGAAGATCACTTGTGTCACTGCACTCCAACCTGGACAACAGAGTGGGACCCTGTCTCAAAAACAAAAACAAACAAACAAAACAGATATAAACATACTAGATAGGACAAGGGGCATGAGAAGAACACACAAAATGGGGGAGTCTCTGTACTTCGCATACCTTCAACGGCATCATACCAATCTCAGTGACGCAGGAAGCAAGGTCATCAGCGGAGATGAGCATCGTGGAGGAGACAATGGTGTGCAGAGGTAGGAGACGCGAGAGGGGGGTGCACTAGATGGAGGAAGCTGCAGGTGTCATAAAGGTCCCCTCAGGCTCATGGCCATGAATTCAAAGGGAGTCAGTGAGTGTGTTTTTCTTCTGACACAATCAGCTGAACAGATGCAGGCAAAGCGTTGATAGAAAATTGGGTTTGCCCAGGGTTATGGCTCTGCCAAGACAGTTGTAGGAAGCGACAGAACCACGAGGAAACTGAGCAGGTGTGAGAGGGATGAGAACCCCTGACGAGGGAAGGGATCAGATCAGAGCAGAGAAGACAGAGGGAAGTGTAGGCTGTAGCTCAGTTTGGAGCTGTGGTAAAGACAAGTGATCCTGGAGTCAAATGCCGGA

First switch

CGGGGGAAGGGCGGTGGAAAGGCCTGGCAGGTTCCAACATTGGAGAAAGATGGAAAGAAATCTCAACTAT CONTIG

CGGGGGAAGGGCGGTGGAAAGGCCTGGCAGGTTCCctattttaatct LEFT 2:2,685,870

ttttattgggAACATTGGAGAAAGATGGAAAGAAATCTCAACTAT RIGHT-REV 2:24,839,259

Second switch

GAAAAGGGAAATGTGCAAATAGC**AG**CTGAGATCGCACCACTACACTCCAG CONTIG

GAAAAGGGAAATGTGCAAATAGC**AG**acacaactcat LEFT REV 2:24,839,100

gttgcagtg**AG**CTGAGATCGCACCACTACACTCCAG RIGHT 17:73,563,667

Third switch

GGAGGCCAAGGTGGGAAGATCAC**TTG**TGTCACTGCACTCCAACCTGGACA CONTIG

GGCCAAGGTGGGAAGATCAC**TTG**agcccag LEFT 17:73,563,807

tgagctgtga**TTG**TGTCACTGCACTCCAACCTGGACA RIGHT 14:34,515,745

Fourth switch (Possible micro-insertion of highlighted bases)

CCTTCAACGGCATCATACCAATCTCAGTGACGCAGGAAGCAAGGTCATCA CONTIG

CCTTCAACGGCATCATggcccttcttccctagg RIGHT REV 14:34,515,904

ctattttaatctTCTCAGTGACGCAGGAAGCAAGGTCATCA LEFT 2:2,685,883

| Switch | MH bases | # of MH bases |
| --- | --- | --- |
| Switch1 | -- | 0 |
| Switch2 | AG | 2 |
| Switch3 | TTG | 3 |
| Switch4 | - | 0 |

**Supplemental Figure S5:** **Genotyping analysis results for the four clonal SVs.** The X- and Y- axes represent respectively the number of reads supportin alternate and reference alleles. For each SV, the clones in which the event was detected had alternate allele supporting reads, while the remaining clones only had reference allele supporting reads. We did not find evidence for these SVs in any of the corresponding tissue or bulk samples. Given the kb-scale length of SVs, number of supporting reads can be higher than sequencing coverage per base.

**Supplemental Figure S6: Complex clonal SV on chr2 detected in brain 275 clone #3 from PA.** The pink segment indicates deletion and yellow indicates inversion. Assembled contig and sequence microhomologies were as below:

>Assembled Contig (reverse compliment)

TCTCTACTAAAGATACAAAAAATTAGCGGGGTGTGGTGGCAGGCGCCTGTAATCCCACTTACTCAGGAGGCTGAGGCAGGAGAGTTGCTTGAACCTGGGAGGCAGAGATTGCAGTGAGCCGAGACTCCATCTCAAAAAAAAAAAATTTTTTTTTGTAGTGACAAGGTGTCACTGTGTTGCCAGGGCTGGTCTCAAACTTCTGGGCTCAAGTGATCCTCCCATTTCGGCCTCCCAAAGTGCTAGGATCACAGGCATGAGTCACTGTGCCTGGTCTTCAAGTTGTTATTAAAGCATGTTTACCCACATTATGCACATGGTATAATGGAAAGTATTGTTGTGGAAGTTAGGAGATAGGGATTCTAGCCTAGCTTTTTATTTTTTTGGGACAAGGTCTCACTTTTTCGCCCCAGGCCGAAGTGCAGTTGTGCGAAAGGCTATCAACTTCATCTAGAGGAAATACAAGTATAAGACTTTAATTAAAGACCAAGCTATGTGAGTCATCTGAGATTAATCTAGTTTACTACTTATTAATATAGTGGGTCCTACTAAATTCATTAGATAGTCTTTAAGCTTGCCCTTCTTTAAAACTTCTTCCTTCTGAATAATGTTTCCAATCGTATCTTTCCTATTTGGAGAGGGAAATTTCACAAATAGTCAATAACTTAGTTGTGAAAGAAGAGAAGGGGAGCACGATGCTAGTAGACGCCTAAAAACTGAATTTCTAATAGAATTTGATGGTGTAAGTGTTGATTATGATATTTTTAATGTGGCAGCATTTTAGTATATTTTCCTATTAAATGGCCAAGGTTAAAAATACAAATATCTTTATATTTGTTATTACTTTTCTAAATGAATTGAAAAAAGATTTTTTGCTTGTAGGTACAGTCTGCTGGAGATGACAGAGTACTTGTAATGGGTGCAACTAATAGGCCACAAGAGCTTGATGAGGCTGTTCTCAGGTAGGGAGATTTATATGGAAATACATGCATTTATTACAGACAATATTTACTCATGTGTCCATCTTACATATTATTTCCTTACTCTCAGTTTTAAGACTAAATTCACTATTTTCTTCTAGTACTATCTCTAGCCACTTGTTACCAACTACATAAGGATTTTGAGGTCAACAGCTTGATATCAGGATAATTAGTCTATTAAAGGTTTAGTTACGGTTATAGGCAAATTGGTCAGTTCTTCTGGGTTGCATTAATTTATCTTT

First switch

GTCTCACTTTTTCGCCCCAGGCCGAAGTGCAGTTGTGCG**A**AAGGCTATCAACTTCATCTAGAGGAAATAC CONTIG

GTCTCACTTTTTCGCCCCAGGCCGAAGTGCAGTTGTGCG**A**tctcg LEFT 32,359,829

cacaca**A**AAGGCTATCAACTTCATCTAGAGGAAATAC RIGHT-REV 32,361,963

Second switch

GTCAATAACTTAGT**TG**TGAAAGAAGAGAAGGGGAGCACGATGCTAGT CONTIG

GTCAATAACTTAGT**TG**gaaaatatgtt LEFT-REV 32,361,720

cttttg**TG**TGAAAGAAGAGAAGGGGAGCACGATGCTAGT RIGHT 32,361,973

| Switch | MH bases | # of MH bases |
| --- | --- | --- |
| Switch1 | A | 1 |
| Switch2 | TG | 2 |

**Supplemental Figure S7: Complex clonal SV on chr15 detected in brain 316 clone #3 from FR.** The green segments indicate duplicated region and red indicates deletion, while pink indicates the normally replicated region. Assembled contig and sequence microhomologies were as below:

>Assembled_Contig_for_316_FR_cl3

TTTTGCTCTTATAAGGATAACACTATATAATACACATCCTGTGTATAAAGTTTGTAAGCATGCTGCTTATTTCAGGAAGGTAAATTTATAAAAATAGAATTACTAAATCAAAATGCATGTATATTCTTGGCTCTTGACCTTTTGACAATAGTTTTAAAAATTGCTTTGAAGATATTTTTG

ATAATTTACACTTCCACCTATAGATAAGACAACCATATCTGGACAAGTATTATTAAATAATTTTATGTATCATTGCCCTTTTGTTAGGAAATAAAAAAGTTATTGCACTCTTTTTTTAACCTGTTTCCTCTTTAATCTACTGTTCAGTTGAAAATATATTCATTAAACAATTGTTTATTG

AACACTTACAGCCCTTCATATGCAAAGTTTCTGTAATATCTTTTGGATATTCAGTTAGAATGTTGACAATTTTGTGCACTGGTTTTTAGTAACTCTCCAAGGTTTCTTTATGAACATGATGTCCTCCTATTCTCTTAAAGTAAAGGTTGCATCAGTTGCAGGGACCTAGAAGTAAAAGTG

AAAAGTCTGCTAGTAAATATGTTTCCCATTTTGTCATTTGTGTCATTTTTTTCTTAGGGTTATGTAAAATAATAAAATTGTATATATTTGTATACTATCTGTACTTTTTCCACTTGTTTTATATATTAGCAATTATTTCTCTATCCTGAGACTAAATAACACAATTTCTCTTAAGTCTCA

GGTTAGTGGAAGAATTACTCCATGTATCACTGCAATACAAATATTTAACTTACTTTGGAAATAATATTTGATGTTTGTAAGATGAATCTTAGAATAATAGGTCACTTTTCCATGTATTTGAAATGCTTTCACTATTATATTGCAGGTTGAAACACACACACACACGTACACACACAAGCA

GACAGAAATACCTCTACTTTAGAATCATAAACCCTTTTTAAAAATAATTCAAAATATATATTCCCTGATAACTTCTCATTTTAATTATTGTGGTTTTATATTTTAATATATTGTAAAGTAAGTATGCTTCTATTACTGTAGTTTTTCAAATGTCTTACTTATT

First switch

TTTGTGCACTGGTTTTTAGTAACTC**TC**CAAGGTTTCTTTATGAACATGATGTCCTCCTAT CONTIG

TTTGTGCACTGGTTTTTAGTAACTC**TC**tgaatataaataa RIGHT 98,098,722

acgttcgaga**TC**CAAGGTTTCTTTATGAACATGATGTCCTCCTAT LEFT 98,097,665

Second switch

AGTAAAAGTGAAAAGTCTGCTA**G**TAAATATGTTTCCCATTTTGTCATTTG CONTIG

AGTAAAAGTGAAAAGTCTGCTA**G**aactcacaaatt LEFT 98,097,760

tttggtttact**G**TAAATATGTTTCCCATTTTGTCATTTG RIGHT 98,098,757

| Switch | MH bases | # of MH bases |
| --- | --- | --- |
| Switch1 | TC | 2 |
| Switch2 | G | 1 |

**Supplemental Figure S8: Distribution of various SV types resulting from all-to-all comparisons in fetal and adult brain samples.**

A.,


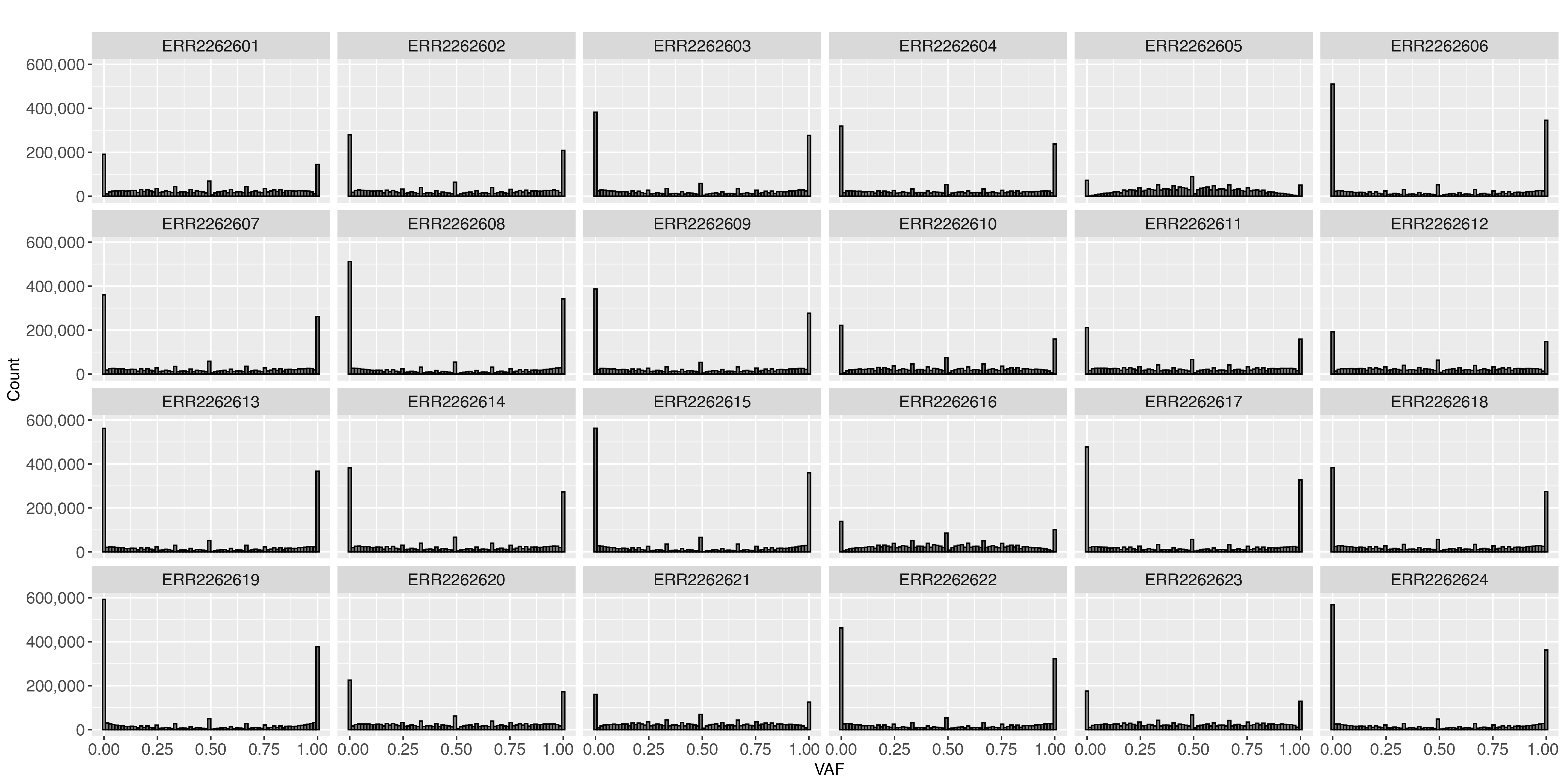


B.


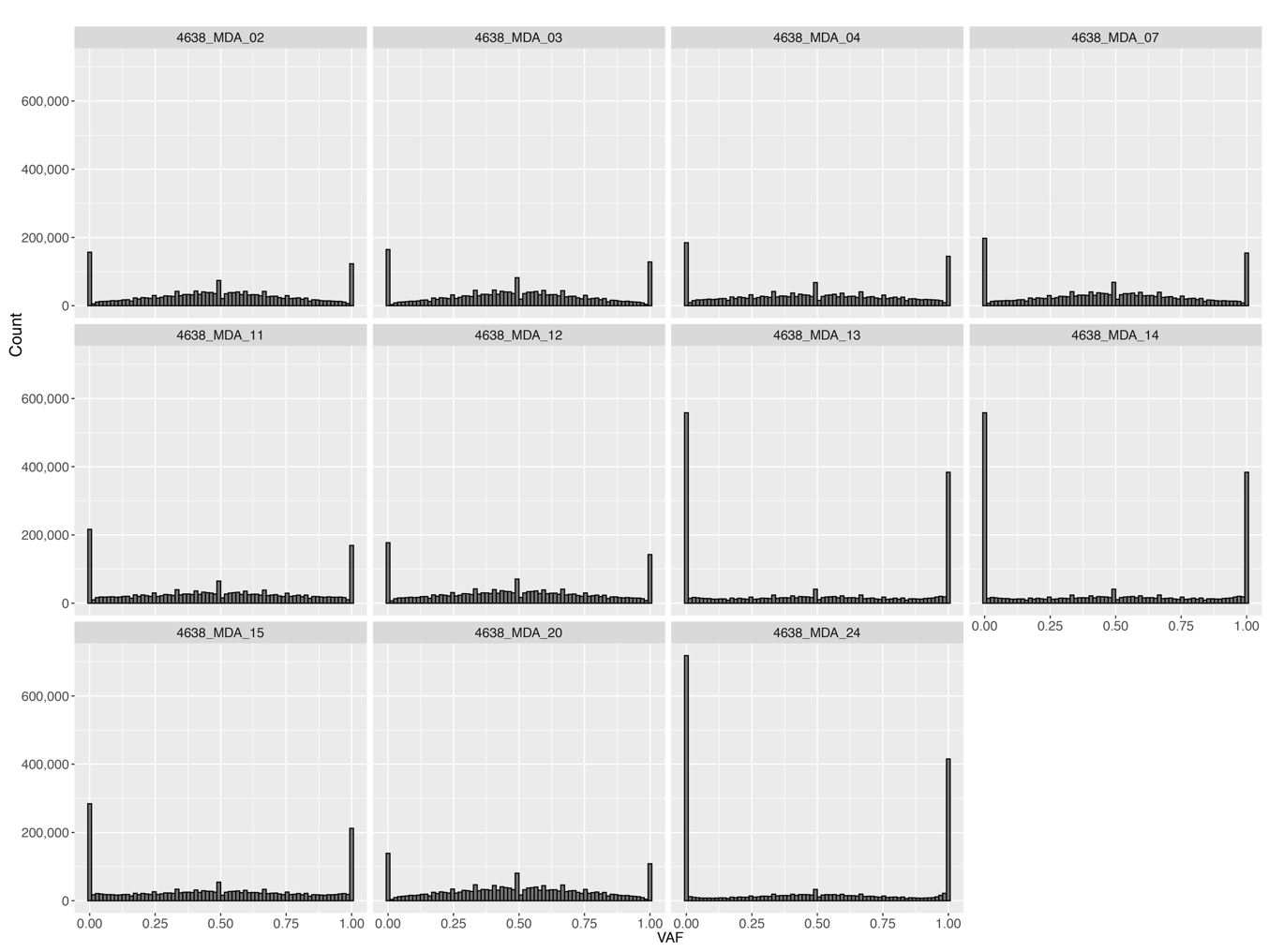


C.

**
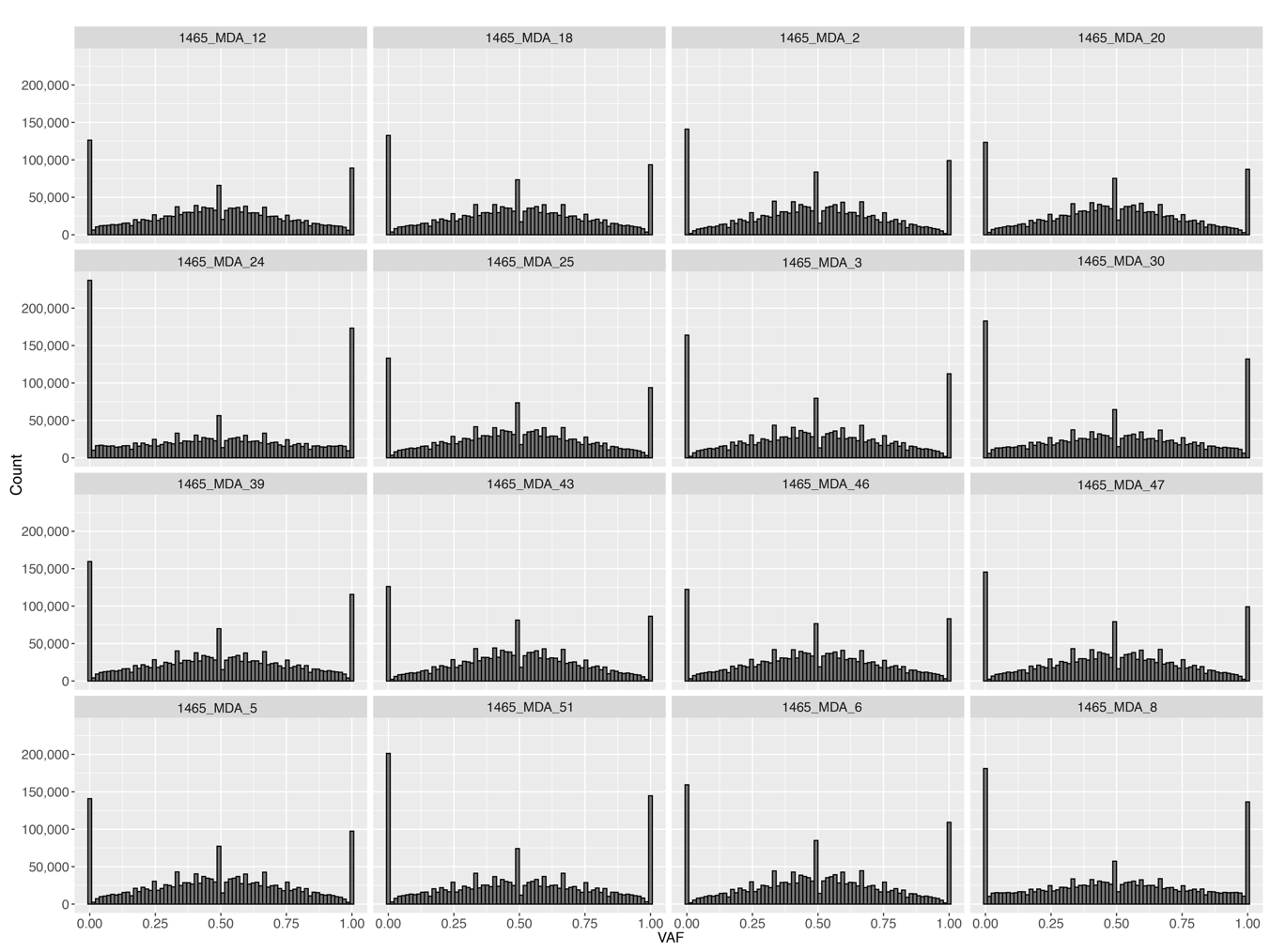
**

D.


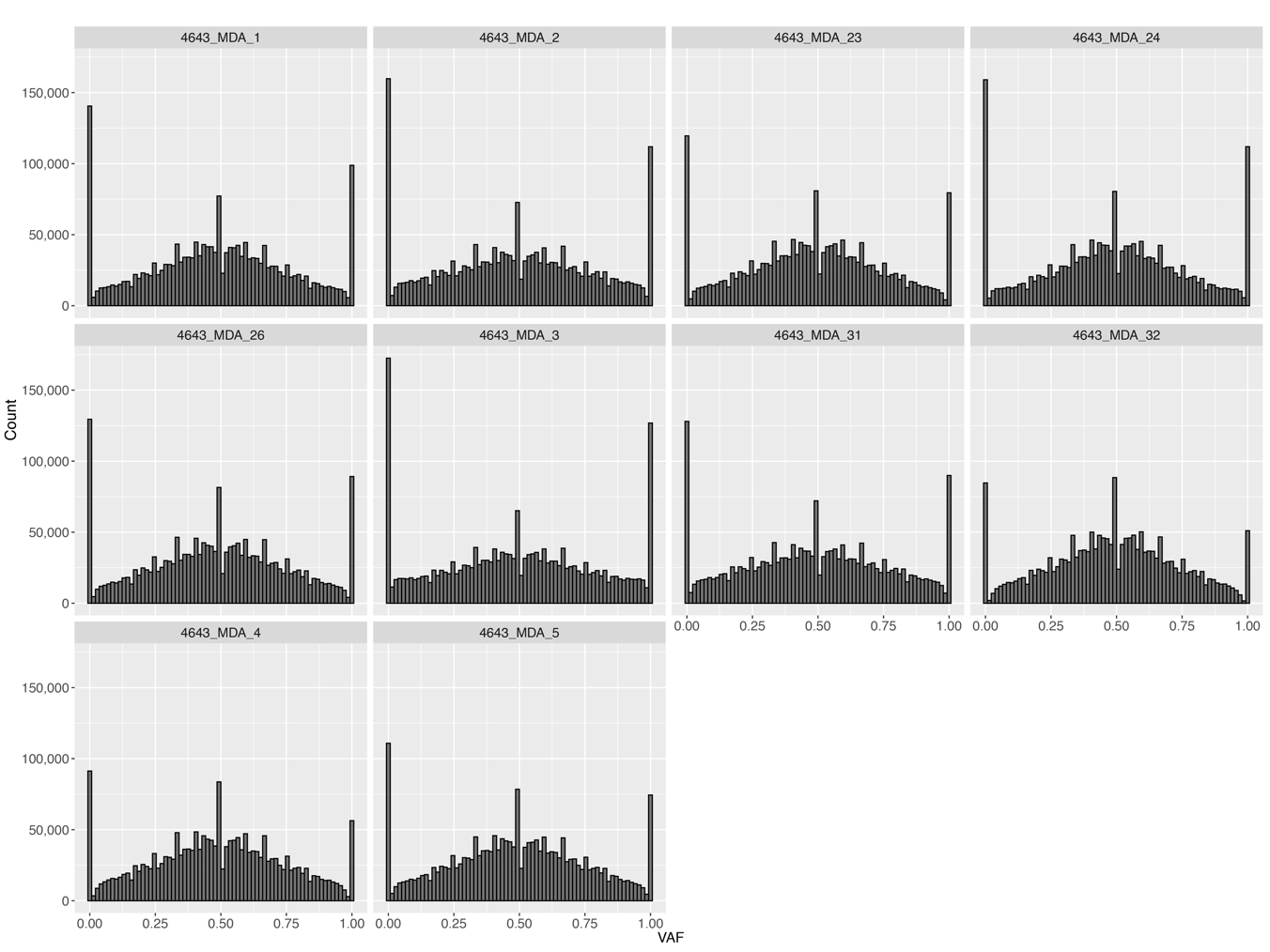


**Supplemental Figure S9**: **Variant allele frequency distribution of germline heterozygous SNPs from single nuclei data.** These neuronal nuclei were isolated from A. hippocampus and B, C, D. pre-frontal cortex from adult post-mortem brains.


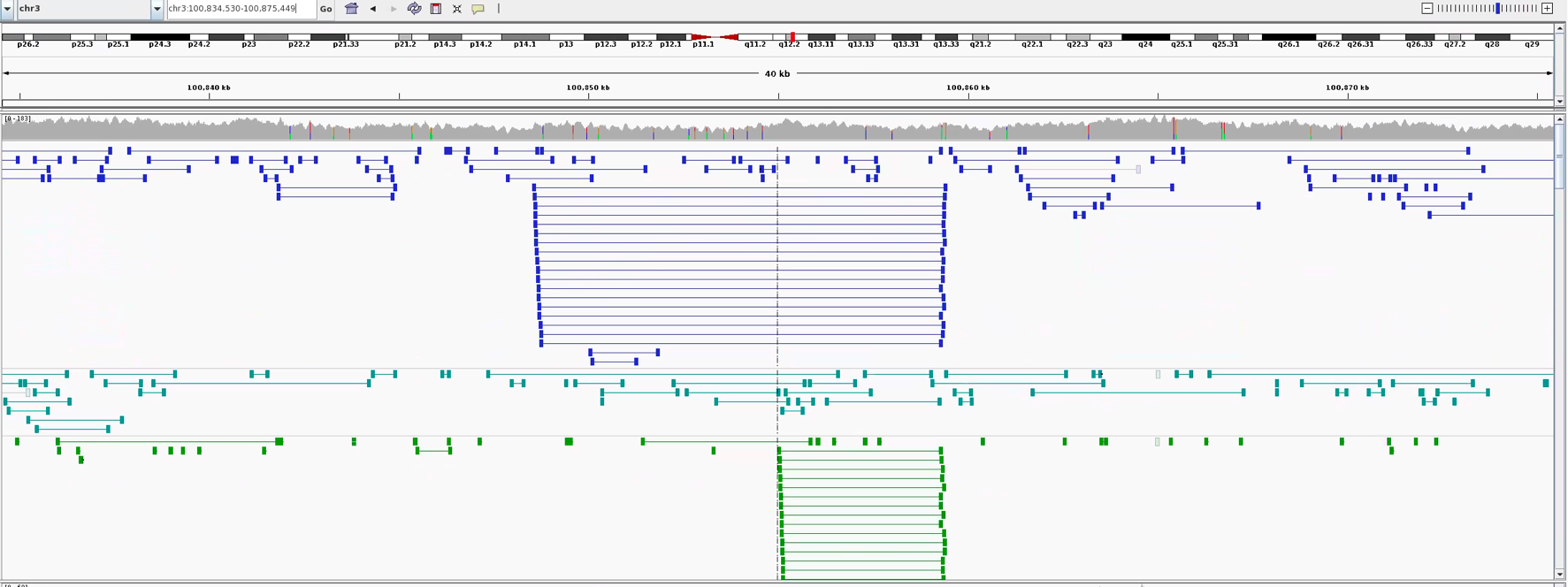


*Brain B, neuron 12*

**Supplemental Figure S10**: **Example of a one-sided inversion and duplication event detected systematically in multiple neurons.** These SVs were not supported by our assembled contigs either, so we considered them as possible amplification artefacts or other false positives and filtered them out. The green reads support duplication, while blue and cyan reads support inversion.


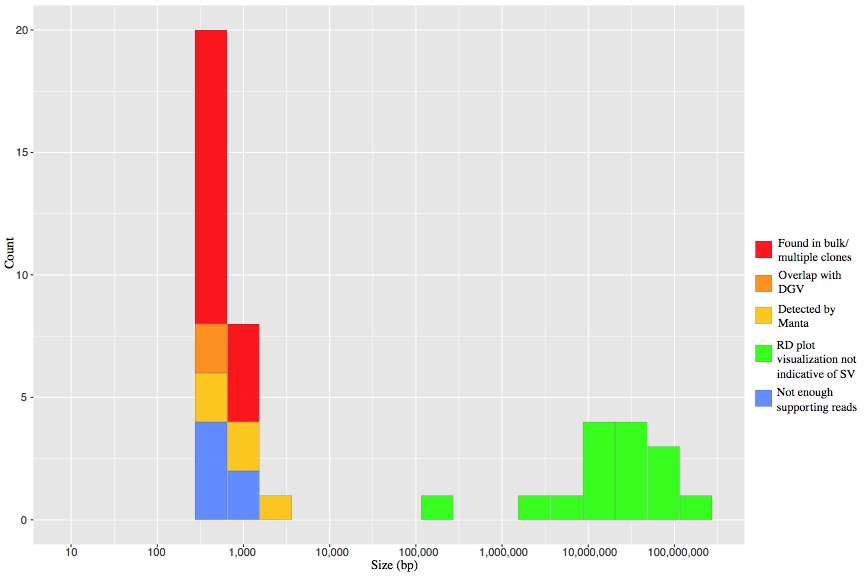


**Supplemental Figure S11: Size distribution of SV calls from DELLY.** Upon visual inspection of SV calls from DELLY, we observed that among the 44 SVs classified as somatic following clone-to-clone comparisons, 16 were found in bulk and/or in multiple clones (size range ~500-1340 bp), 15 (called as deletion or duplication) showed no difference in read depths based on their read depth plot visualization using CNVnator (size range ~215,000-133,000,000 bp) and showed no complexity around breakpoints, 6 had < 4 supporting reads (possibly sub-clonal), 2 overlapped with variants from DGV and the remaining 5 were part of 2 complex SVs also detected by Manta.

| **Coordinates** | **Clone** | **Conclusions** | **Absolute size = Len(Ref)-Len(Alt)** | **Size of segments** | **Size of jumps** | **Size of deleted regions** |
| --- | --- | --- | --- | --- | --- | --- |
| chr2:2,685,870-2,685,883 | 320-FR-cl4, FR-cl8 | clonal | 286 | 159 + 140 + 159 = 458 | interchromosomal | 13 |
| chr15:98,097,662-98,098,720 | 316-FR-cl3 | clonal | 35 | 95 + 962 = 1,057 | 1,057 + 997 | 35 |
| chr2:32,360,001-32,361,600 | 275-PA-cl3 | clonal | 1901 | 243 | 2,134 + 253 | 1891 + 10 |
| chr12:78,381,480-78,383,762 | 316-BG-cl19 | clonal | 828 | 327 + 385 + 204 = 916 | 350 + 546 + 644 + 1034 | 23 |
| chr5: 130,150,883-130,152,883 | 320-FR-cl19 | sub-clonal | 1811 | 165 + 23 = 188 | 2000 + 1409 + 1573 |  |
| chrX:148,472,606-148,472,843 | 316-BG-cl20 | sub-clonal | 237 |  |  |  |
| chr10:16,611,028-16,611,440 | 316-BG-cl18 | sub-clonal | 412 |  |  |  |
| chr15:60,605,351-60,606,081 | 320-FR-cl6 | sub-clonal | 730 |  |  |  |
| chr13:43,237,119-43,238,168 | 275-FR-cl12 | sub-clonal | 1049 |  |  |  |
| chr9:14,568,175 -14,573,784 | 320-FR-cl12 | sub-clonal | 5609 |  |  |  |

**Supplemental Table S1**: Summary of clonal and sub-clonal events detected using our custom SV detection workflow summarized in Fig. 1A.
